# Comparison of maternal isocaloric high carbohydrate and high fat diets on osteogenic and adipogenic genes expression in adolescent mice offspring

**DOI:** 10.1186/s12986-016-0130-x

**Published:** 2016-10-18

**Authors:** Seyedeh Neda Mousavi, Fariba Koohdani, Farzad Shidfar, Mohamadreza Baghaban Eslaminejad

**Affiliations:** 1 Department of Cellular and Molecular Nutrition, School of Nutritional Sciences and Dietetics, Tehran University of Medical Sciences, Tehran, Iran; 2 Department of Nutrition, School of Health, Iran University of Medical Sciences, Tehran, Iran; 3 Department of Stem Cells and Developmental Biology, Cell Science Research Center, Royan Institute for Stem Cell Biology and Technology, ACECR, Tehran, Iran; 4 Department of Biochemistry and Nutrition, School of Medicine, Zanjan University of Medical Sciences, Zanjan, Iran

**Keywords:** Osteoblastogenesis, Adipogenesis, Macronutrients, Isocaloric diet, Mice

## Abstract

**Background:**

Maternal high fat/high calorie diet leads to adiposity and bone fracture in offspring. However, the effects of macronutrient distribution in maternal isocaloric diet have not been studied. The present study was designed to test the hypothesis that maternal isocaloric pair-fed high-carbohydrate diet will increase osteoblastic and decrease osteoclastic and adipogenic gene expression compared with high-fat diet in adolescent mice offspring.

**Methods:**

Virgin female C57BL/6 mice were impregnated and fed either the AIN 93G isocaloric pair-fed high-carbohydrate (LF-HCD) or a high fat (HF-LCD) diet from the time of vaginal plug confirmation until the offspring was weaned.

**Results:**

After adjusting for the sex of offspring, osteoprotegrin (OPG) and Ctnnb1 (beta-catenin) genes expression were significantly reduced by 98 % and 97 % in the bone of offspring born from the HF-LCD compared with the LF-HCD-fed mothers (*p* < 0.001 and *p* < 0.001, respectively). Peroxisome proliferator-activated receptor gamma-2 (PPAR γ2) gene expression in the bone of offspring born from the HF-LCD was 7.1-folds higher than the LF-HCD-fed mothers (*p* = 0.004). In the retroperitoneal fat mass of offspring born from HF-LCD, AdipoQ and LPL genes expression were respectively up-regulated 15.8 and 4.2-folds compared with the LF-HCD-fed mothers (*p* < 0.001 and *p* = 0.03, respectively).

**Conclusions:**

Maternal isocaloric pair-fed high-carbohydrate diet enhances osteoblastogenesis and inhibits adipogenesis compared with high-fat diet in adolescent mice offspring.

## Background

Obesity and osteoporosis have increased globally [1, 2]. Adipose tissue is an endocrine gland that affects bone metabolism [3]. In recent years, the importance of the association between fat and bone in the pathophysiology of bone loss has been highlighted [4]. Osteoblasts and adipocytes are derived from a common multipotential mesenchymal stem cell (MSC) progenitor [5]. Bone is constantly remodeled throughout the process of bone formation by osteoblasts and bone resorption by osteoclasts [6]. Developmentally, osteoclasts are derived from hematopoietic stem cell precursors of the monocyte/macrophage lineage in the blood and the bone marrow, while osteoblasts originate from bone marrow mesenchymal stem cells [7]. Considerable evidence exists to support that a shift in MSC differentiation to favor the adipocyte over the osteoblast lineage can directly contribute to imbalances in bone formation/resorption and ultimately leads to bone loss. This shift of MSC differentiation to the adipocyte lineage may contribute to the progressive increase in adipocyte formation and decrease in osteoblast number that coincides with age-related bone loss [8]. Adipocytes may further influence on bone remodeling through the secretion of adipokines with paracrine actions that may influence the development and function of stem cell precursors as well as mature cell types such as osteoblasts and osteoclasts [9]. Thus, given the close association between adipocyte and osteoblast formation, the potential exists to prevent or treat bone loss by inhibiting bone marrow adipogenesis.

Unbalanced feeding is one of the most determinant factors that can induce metabolic disorders [10]. In this context, a number of experimental studies have demonstrated that not only the energy intake, but also the macronutrient distribution of the diet may play an important role in obesity, diabetes, hypertension and other metabolic disorders [11, 12]. Macronutrient distribution in maternal diet during pregnancy may affect body composition of the offspring later in life, but evidence is still scarce [13]. Evidence from experimental models clearly demonstrate that maternal obesity, independent of birth weight, leads to developmental programming of adiposity gain in the offspring [14, 15]. Unfortunately there are currently no explanations, either clinically or experimentally, to assess the macronutrient composition in maternal isocaloric diet during gestation and lactation on osteoblastic, osteoclastic and adipogenic genes expression in adolescent mice offspring. Therefore, this study was designed to investigate the different effects of maternal isocaloric low fat-high carbohydrate diet (LF-HCD) and high fat-low carbohydrate diet (HF-LCD) on osteoblastic, osteoclastic and adipogenic genes expression in adolescent mice offspring. We hypothesized that isocaloric pair-fed HF-LCD induces osteoclastogenesis and adipogenesis compared with LF-HCD in adolescent offspring. To achieve this, virgin female C57BL/6 mice were used as a model to study the effects of two isocaloric diets containing different amounts of fat and carbohydrate during gestation and lactation on bone osteoblastic, osteoclastic and adipogenic gene expression levels in adolescent male or female offspring.

## Methods

The experimental protocol was approved by the Animal Research Committee of Iran University of Medical Sciences (protocol number: 24208). This research conforms to the Institutional and National Guide for the Care and Use of Laboratory Animals. Eight-week-old inbred female C57BL/6 mice (21 ± 1.5 g) were obtained from the Razi Vaccine and Serum Research Institute, Tehran, Iran. Each mouse was individually housed at 21–23 °C with controlled humidity (50 ± 5 %) under a 12 h artificial light cycle (7 am to 7 pm). The AIN 93 M diet composition (per 1 kg) was 140 g of protein as Casein lactate (Iranian Caseinate Industry, Iran) and 1.8 g as L-cystein (W326305, Sigma Aldrich, Germany), 40 g of lipid as soybean oil (Kamzit Company, Iran), 630 g of carbohydrate as corn starch (corn dextrin from corn refinery, Iran), 100 g as sugar (local confectionery, Iran), 35 g of AIN 93 M mineral mix (296040002, MP Biomedicals, USA), 10 g of AIN 93 vitamin mix (296040201, MP Biomedicals, USA), 2.5 g of choline bitartrate (C1629, Sigma Aldrich, Germany), 0.008 g of tert-butyl hydroquinone (112941, Sigma Aldrich, Germany) and 50 g of fiber (wheat bran, Iran).

**Fig. 1:**
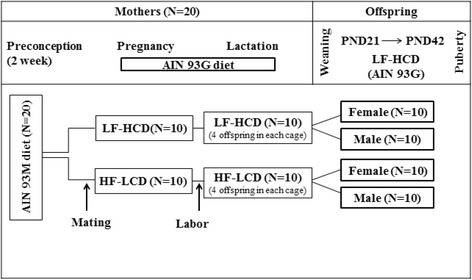
Experimental timeline. LF-HCD: low fat-high carbohydrate diet; HF-LCD: high fat-low carbohydrate diet

Experimental timeline was shown in Fig. 1. Each female mouse was mated with one male per cage overnight. After vaginal plug confirmation, mothers were randomly assigned to dietary groups (*N* = 10 in each group), as shown in Table 1. The LF-HCD is considered as the control diet group.

**Table 1:** Composition of the experimental diets per 1 kg during the study (AIN 93G diet)

Diets	LF-HCD	HF-LCD
Nutrients (g/kg)		
Casein	200	200
Cornstarch	530	247
Sucrose	100	100
Soy oil	70	198
Fiber	50	204.5
Mineral mix	35	35
Vitamin mix	10	10
L-cys^a^	3	3
Choline bitartrate	2.5	2.5
*tert*-butyl hydroquinone	0.008	0.008
Energy (kcal/gr)	3.97	3.97

*LF-HCD* low fat-high carbohydrate diet, *HF-LCD* high fat-low carbohydrate diet

^a^L- cysteine

During the study, all animals were isocalorically fed with the same kcal/ [g body weight] of the group that ate less (*pair-fed* model) [16]. Offspring were reduced to 4 pups in each cage, nursed by birth mothers and weaned on day 21, when they were separated according to sex with a maximum of four mice per cage. Pups received ad libitum control diet post-weaning. One male and one female offspring were randomly selected from each cage for final gene expression analysis at 6 weeks of age.

### Weight and biochemical measures

Animals were weighed on a calibrated balance scale (Marte Scale (EK-3000i, USA)) at birth and adolescence. All pups were killed at 6 weeks of age by administration of 40 mg/kg ketamine and 8 mg/kg xylazine, following the overnight fasting conditions. Blood samples were collected at 08.00 a.m., after overnight fasting from the heart. All the biochemical parameters were measured by autoanalyzer. Serum OPG (R&D system; Cat ≠ DY459), RANK-L (R&D system; Cat ≠ MTR00), Lep (R&D system; Cat ≠ MOB00) and AdipoQ (EMD Millipore; Cat ≠ EZMADP-60 K) were measured by ELISA method, according to the manufacturer’s instructions.

### Collection and preparation of specimens

The right femur and retroperitoneal fat were dissected out, stripped of soft tissue using a scalpel, immediately frozen and then were powdered.

### RNA isolation and real-time polymerase chain reaction (RT-PCR)

Total RNA from the powdered samples were extracted using QIAzol Lysis Reagent (QIAGEN Inc., Valencia, CA 91355, USA) according to the manufacturer’s instructions, followed by DNase digestion and column cleanup using RNeasy mini columns (Qiagen, Valencia, CA, USA) [17]. RNA quantification was assessed at 260 nm with a ND-1000 spectrophotometer (NanoDrop, Wilmington, DE, USA), and its quality (integrity) was checked by agarose gel electrophoresis. One microgram of total RNA was retro-transcribed into cDNA using Prime-Script cDNA synthesis reagents from Takara (Takara Bio, Inc., Japan) in 20 μl volume. The RT-PCR was performed in an ABI StepOne sequence detection system (Applied Biosystems, California, USA) with 1 μl of cDNA and 10 pmol of each primer corresponding to the tested genes, using the SYBR Green I Master Mix (Roche). The expression assay was run in duplicate. Primer pairs used were shown in Table 2.

**Table 2:** Real-time-PCR primer sequences

Gene	Forward primer	Reverse primer
*OPG*	5′-GGGCGTTACCTGGAGATCG-3′	3′-CGTTGTCATGTGTTGCATTTCC-5′
*RANKL*	5′-CAGCATCGCTCTGTTCCTGTA-3′	3′-CTGCGTTTTCATGGAGTCTCA-5′
*Ctnnb1*	5′-CCTCCCAAGTCCTTTATGAATGG-3′	3′-CCGTCAATATCAGCTACTTGCTCTT-5′
*PPARγ2*	5′-GCCCTTTGGTGACTTTATGGA-3′	3′-GCAGCAGGTTGTCTTGGATG-5′
*LPL*	5′- GGGAGTTTGGCTCCAGAGTTT-3′	3′- CGTGTGTGAAATGTCATTGATCC-5′
*Lep*	5′- GTGGCTTTGGTCCTATCTGTC-3′	3′- CGTGTGTGAAATGTCATTGATCC-5′
*AdipoQ*	5′- TGTTCCTCTTAATCCTGCCCA-3′	3′- CCAACCTGCACAAGTTCCCTT-5
*FASN*	5′- AGAGATCCCGAGACGCTTCT-3′	3′-GCTTGGTCCTTTGAAGTCGAAGA-5′
*Runx2*	5′- CAGCATCCTATCAGTTCCCAA-3′	5′- CAGCGTCAACACCATCATT-3′
*GAPDH*	5′- GACTTCAACAGCAACTCCCAC-3′	3′- TCCACCACCCTGTTGCTGTA-5′

*OPG* osteoprotegrin, *RANK-L* Receptor activator of nuclear factor kappa-B ligand, *Ctnnb1* beta-catenin, *PPAR γ2* Peroxisome proliferator-activated receptor gamma-2, *LPL* lipoprotein lipase, *Lep* leptin, *AdipoQ* adiponectin, *FASN* fatty acid synthase, *Runx2* Runt-related gene 2, *GAPDH* glyceraldehyde 3-phosphate dehydrogenase

All primers for RT-PCR analysis were designed using Primer Express software 2.0.0 (Applied Biosystems). The relative quantity of mRNA was calculated for each sample using the copy threshold (Ct) value and normalized against glyceraldehyde 3-phosphate dehydrogenase (GAPDH, housekeeping gene) mRNA. The stability of the housekeeping gene was considered a Ct standard deviation <1 and was therefore considered an appropriate control [18]. The amplification profile included one cycle at 95 °C for 10 min and 40 two-step cycles: 95 °C for 15 s and 60 °C for 60 s. The results were generated and analyzed using the 2^^-∆∆Ct^ method as described by Livak and Schmittgen [19].

### Statistical analyses

In the designing of the study, to detect a difference in total body fat mass between two groups, we used a 90 % confidence interval with a two-sided test with α =*0.05* (type I error). On the basis of SDs reported in a similar study [20], the number of subjects needed to detect this difference was 16 per group. Data were tested for normal distribution using the Kolmogorov-Smirnov test. Independent sample *t*-test was used to compare the variables between two groups. Data did not have normal distributions for gene expression even after all of the transformation methods. The differences between two variables were measured by the Mann–Whitney Wilcoxon test, controlling for the sex of offspring. To assess the interactions between sex and diets on the target gene expression, a logistic regression model was used in which any gene was coded as a nominal variable (gene expression above the 50th percentile or below the 50th percentile) [21]. All data are expressed as the means ± SE. The level of significance was set at *P* < 0.05. Statistical analyses were performed with the IBM SPSS Statistics software (version 18; IBM Corp).

## Results

The average of food intake was not significantly different between the LF-HCD versus HF-LCD group at the end of weeks 1 (2.4 ± 0.17 g/day vs. 2.44 ± 0.2 g/day), week 2 (2.99 ± 0.18 g/day vs. 2.92 ± 0.12 g/day) or week 3 (5.94 ± 0.27 g/day vs. 5.91 ± 0.2 g/day), post-weaning. In the female offspring, adolescence weight was significantly higher in the LF-HCD than HF-LCD group (*p* < 0.001). Serum HDL.C was significantly higher in the HF-LCD than LF-HCD group (*p* = 0.006). In male offspring, there were significant differences in birth and adolescence weight (*p* = 0.008 and *p* = 0.01, respectively). Also, serum glucose was significantly higher in male than females in the LF-HCD group (*p* = 0.005). In the HF-LCD group, there were significant differences in adolescence weight, TC, LDL.C and TG between males and females (*p* < 0.001, *p* = 0.008, *p* = 0.01 and *p* = 0.002, respectively) (Table 3).

**Table 3:** Weight and biochemical measures in the male and female offspring of dietary groups^a^

Variables	Sex	LF-HCD	HF-LCD	*p* value
Birth weight (g)	Female	1.29 ± 0.31^b^	1.3 ± 0.13	0.9
	Male	1.7 ± 0.23	1.3 ± 0.12	<0.001^*^
Adolescent weight (g)	Female	19.7 ± 1.06^b^	17.4 ± 1.4^c^	0.002^*^
	Male	21.3 ± 1.3	21.8 ± 0.79	0.3
TC (mg/dl)	Female	177.1 ± 61.4	146.1 ± 16.5^c^	0.2
	Male	207.5 ± 81.3	205.4 ± 51.9	0.9
LDL.C (mg/dl)	Female	93.3 ± 51.5	54.7 ± 19.9^c^	0.08
	Male	111.2 ± 65.6	89.9 ± 37.05	0.4
HDL.C (mg/dl)	Female	31.9 ± 10.4	48 ± 9.6	0.006^*^
	Male	42.8 ± 11.6	49.2 ± 18.9	0.4
Triglyceride (mg/dl)	Female	179.6 ± 97.4	156.7 ± 24.9^c^	0.2
	Male	266.8 ± 132.8	273.1 ± 69.7	0.9
Glucose (mg/dl)	Female	136 ± 23.6^b^	118.7 ± 33.2	0.2
	Male	183.8 ± 32.8	153 ± 67.7	0.3

*p* values are calculated with the independent sample *t*-test; *TC* total cholesterol

^a^All data reported as mean ± SD; ^b^significant difference between male and female offspring in the LF-HCD group (*p* ≤ 0.01); ^c^significant difference between male and female offspring in the HF-LCD group (*p* ≤ 0.01)

^*^significant differences between LF-HCD and HF-LCD group

**Fig. 2:**
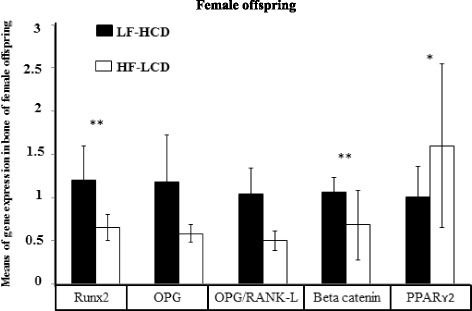
Means of bone genes expression in adolescent female mice offspring. Runx2 and Ctnnb1 gene expression were significantly higher in the LF-HCD than the HF-LCD group. In contrast, PPARɤ2 gene expression was significantly higher in the HF-LCD group (*p < 0.05; **p < 0.001)

**Fig. 3:**
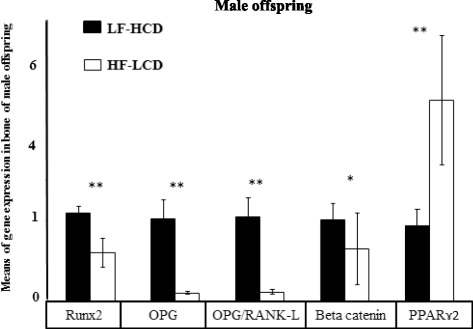
Means of bone genes expression in adolescent male mice offspring. Runx2, OPG, OPG/RANK-L and Ctnnb1 genes expression were significantly higher in the LF-HCD than the HF-LCD group. In contrast, PPARɤ2 gene expression was significantly higher in the HF-LCD group (*p < 0.05; **p < 0.001)

**Fig. 4:**
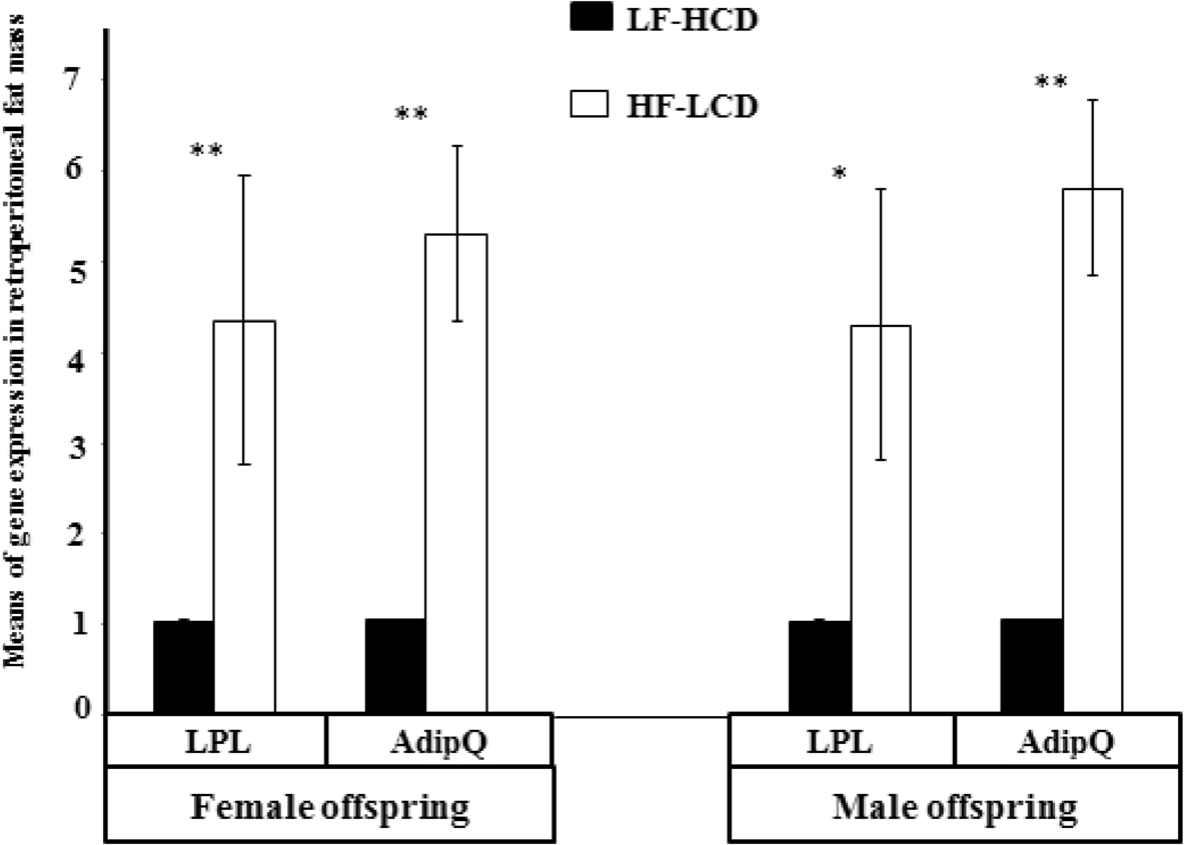
Means of LPL and AdipoQ genes expression in adolescent female and male mice offspring. LPL and AdipoQ genes expression were significantly higher in the HF-LCD than the HF-LCD group (*p < 0.05; **p < 0.001)

**Fig. 5:**
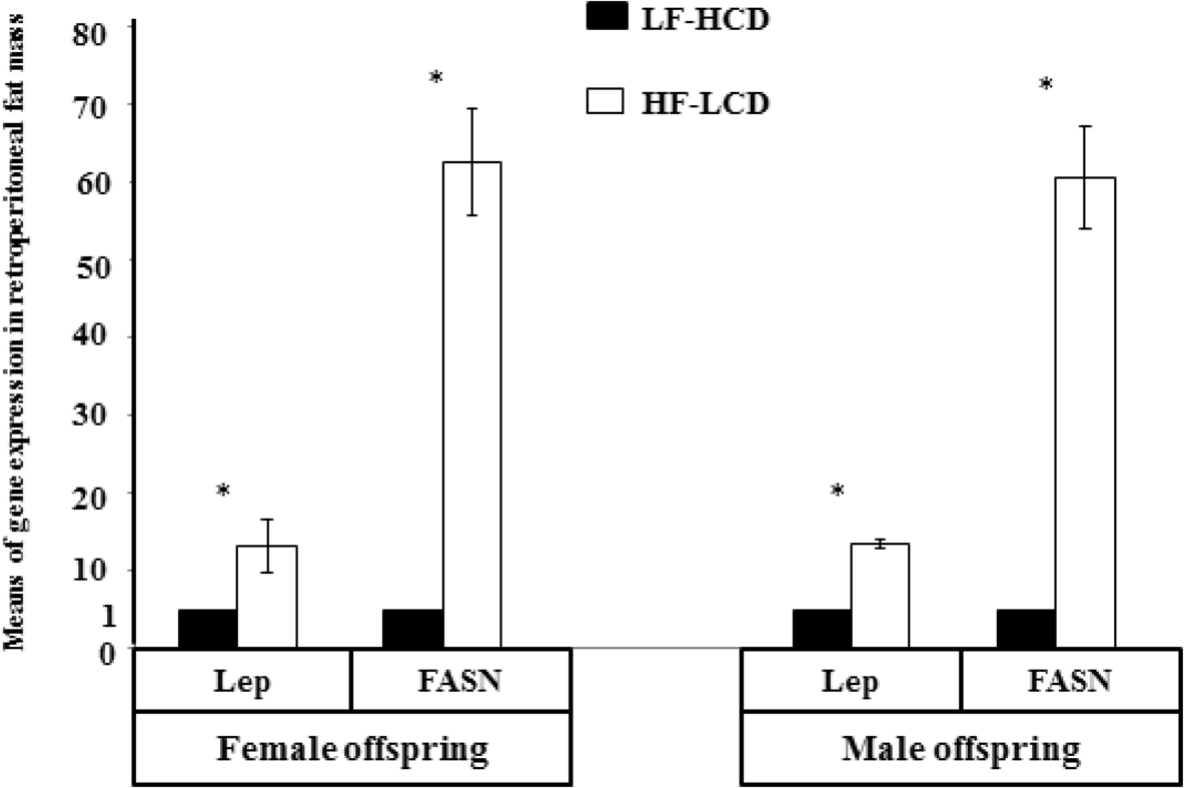
Means of Lep and FASN genes expression in adolescent female and male mice offspring. Lep and FASN genes expression were significantly higher in the HF-LCD than the LF-HCD group (*p < 0.05; **p < 0.001)

As shown in Figs. 2 and 3, Runx2 and Ctnnb1 gene expression were significantly higher in female offspring born from the LF-HCD compared with the HF-LCD-fed mothers (*p* < 0.001 and *p* = 0.01, respectively). In male offspring, mRNA expression of Runx2, OPG, OPG/RANK-L ratio and Ctnnb1 were significantly higher in the offspring born from LF-HCD than HF-LCD-fed mothers (*p* < 0.001, *p* < 0.001, *p* < 0.001 and *p* = 0.03, respectively). In contrast, PPARɤ2 gene expression was significantly higher in female and male offspring of the HF-LCD than LF-HCD group (*p* = 0.04 and *p* < 0.001, respectively). LPL, Lep, AdipoQ and FASN genes expression were significantly higher in female, as well as in male offspring born from HF-LCD compared with the LF-HCD-fed mothers (*p* < 0.001, *p* < 0.001, *p* < 0.001 and *p* < 0.001, respectively). (Figs. 4 and 5)

According to the results of the logistic regression model, the diets showed significant effects on OPG, Ctnnb1, PPARɤ2 genes expression in the bone (*p* < 0.001, *p* < 0.001 and *p* = 0.004, respectively), as well as LPL and AdipoQ genes expression in the retroperitoneal fat (*p* = 0.03 and *p* < 0.001, respectively), after being adjusted for the sex of offspring. OPG and Ctnnb1 genes expression were significantly reduced by 98 % and 97 % in the bone of offspring born from the HF-LCD compared with the LF-HCD-fed mothers. PPARɤ2 gene expression in the bone of offspring born from the HF-LCD was 7.1-folds higher than the LF-HCD-fed mothers. In the retroperitoneal fat mass of offspring born from HF-LCD, AdipoQ and LPL genes expression were respectively up-regulated 15.8 and 4.2-folds compared with the LF-HCD-fed mothers. Adjusting for the diets, the sex of offspring had significant effect on OPG/RANK-L expression ratio (*p* = 0.02). OPG/RANK-L expression ratio was significantly up-regulated by 80 % in the bone of female compared to male offspring (Table 4).

**Table 4:** Status of the target gene expression, diets and sex of offspring

Gene	Variable’s level		B^a^	SE	*p* value	OR (95 % CI)
Runx2	Diets	LF-HCD	-	-	0.9	1
		HF-LCD	−40.6	1.3		0.001 (0.001, 0.001)
	Sex	Female	-	-	0.9	1
		Male	−18.4	9.1		0.001 (0.001, 0.001)
OPG	Diets	LF-HCD	-	-	<0.001^*^	1
		HF-LCD	−3.7	0.95		0.02 (0.004, 0.16)
	Sex	Female	-	-	0.19	1
		Male	1.2	0.89		3.2 (0.55, 18.3)
OPG/RANK-L	Diets	LF-HCD	-	-	0.12	1
		HF-LCD	−1.01	0.65		0.36 (0.1, 1.31)
	Sex	Female	-	-	0.02^*^	1
		Male	−1.6	0.7		0.2 (0.05, 0.78)
Ctnnb1	Diets	LF-HCD	-	-	<0.001^*^	1
		HF-LCD	−3.5	0.88		0.03 (0.005, 0.17)
	Sex	Female	-	-	0.6	1
		Male	0.44	0.82		1.5 (0.31, 7.73)
PPARɤ2	Diets	LF-HCD	-	-	0.004^*^	1
		HF-LCD	1.95	0.68		7.1 (1.83, 27.1)
	Sex	Female	-	-	0.13	1
		Male	1.04	0.69		2.8 (0.73, 11.1)
FASN	Diets	LF-HCD	-	-	0.99	1
		HF-LCD	21.9	817		0.001 (0.001, 0.001)
	Sex	Female	-	-	0.56	1
		Male	0.51	0.87		1.66 (0.3, 9.3)
AdipoQ	Diets	LF-HCD	-	-	<0.001^*^	1
		HF-LCD	2.8	0.74		15.8 (3.7, 67.4)
	Sex	Female	-	-	0.4	1
		Male	−0.62	0.74		0.53 (0.125, 2.3)
Lep	Diets	LF-HCD	-	-	0.99	1
		HF-LCD	40.1	102		0.001 (0.001, 0.001)
	Sex	Female	-	-	0.99	1
		Male	−19.7	72		0.001 (0.001, 0.001)
LPL	Diets	LF-HCD	-	-	0.03^*^	1
		HF-LCD	1.44	0.68		4.2 (1.1, 16.2)
	Sex	Female	-	-	0.87	1
		Male	0.11	0.66		1.1 (0.3, 4.1)

*p* values are calculated with the logistic regression model

*LF-HCD* low fat-high carbohydrate diet, *HF-LCD* high fat-low carbohydrate diet, *OR* odds ratio, *CI* confidence interval, *OPG* osteoprotegerin, *RANK-L* nuclear factor (NF-kB) ligand, *PPRRɤ2* Peroxisome proliferator-activated receptor gamma 2, *FASN* fatty acid synthase, *AdipoQ* adiponectin, *Lep* leptin, *LPL* lipoprotein lipase

^a^Estimated regression coefficients and corresponded standard errors of logistic regression model

^*^significant differences adjusted for maternal diet and/or sex of offspring

**Fig. 6:**
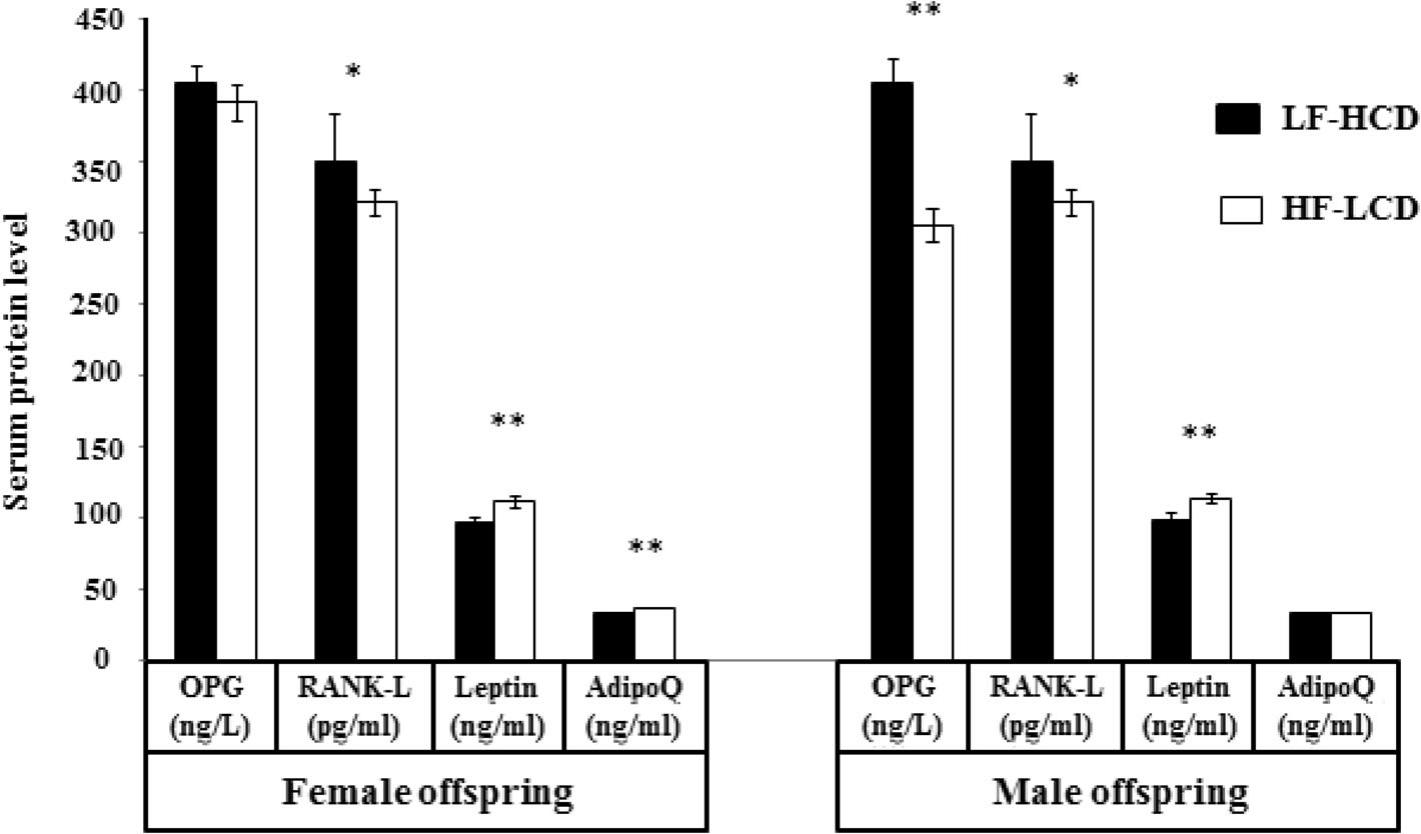
Means of serum OPG, RANK-L, Lep and AdipoQ levels in female and male mice offspring (*p ≤ 0.01; **p < 0.001)

In female offspring, serum level of RANK-L was significantly higher in the LF-HCD than HF-LCD group (*p* < 0.001). But, serum levels of Lep and AdipoQ were significantly lower in the LF-HCD than HF-LCD group (*p* < 0.001 and *p* < 0.001, respectively). In male offspring, Serum level of OPG was significantly higher in the LF-HCD than HF-LCD group (*p* < 0.001), but Lep protein level was significantly lower in the LF-HCD than HF-LCD group (*p* < 0.001) (Fig. 6).

Serum OPG/RANK-L ratio was significantly higher in the male and female offspring of LF-HCD than HF-LCD group (*p* = 0.01 and *p* = 0.02, respectively).

According to the results of the logistic regression model, the diets showed significant effects on serum OPG/RANK-L ratio, adjusted for the sex of offspring (*p* = 0.04). Serum OPG/RANK-L ratio was 5.5-folds higher in the offspring born from LF-HCD than HF-LCD-fed mothers. After adjusting for the diets, serum OPG/RANK-L ratio was significantly higher in the females than males (*p* < 0.001). Serum OPG/RANK-L ratio increased by 96 % in the females than males, adjusted for the diets.

## Discussion

To our knowledge, these are the first results that demonstrate the effects of maternal isocaloric pair-fed high-carbohydrate (LF-HCD) versus high-fat diet (HF-LCD) during gestation and lactation on gene expression and serum levels of formation and resorption markers in bone, as well as adipogenic and lipogenic markers in retroperitoneal fat mass of mice offspring at adolescence. The results of the present study showed that maternal LF-HCD during gestation and lactation lead to up-regulation of Runx2 and Ctnnb1, as well as Runx2, OPG, OPG/RANK-L ratio and Ctnnb1 mRNA expression in bone of female and male offspring, respectively. Also, serum levels of OPG/RNK-L ratio which is the marker of osteogenesis [22] were increased in the LF-HCD-fed group, compared with the HF-LCD. PPARγ2 mRNA expression, as well as other adipogenic genes measured in the current study and serum levels of proteins were increased in the offspring of HF-LCD-fed mothers. Our results showed that mRNA expression of OPG and Ctnnb1 were reduced in the bone of offspring born from the HF-LCD compared with the LF-HCD-fed mothers, adjusted for the sex. In contrast, PPARɤ2 gene expression was increased. Adjusted for the diets, OPG/RANK-L expression ratio was higher in the female than male offspring. Notably also, adjusted for the sex of offspring, serum levels of OPG/RANK-L ratio was significantly increased in the LF-HCD vs. HF-LCD group which result in osteogenesis. OPG and RANK-L are respectively determinants of osteoblastogenesis and osteoclastogenesis, in which OPG can block the effects of RANK-L [23]. Increase in OPG/RANK-L ratio is a determinant of osteoblastogenesis. Runx2 is a transcriptional factor which modulates many aspects of skeletal development [24]. In another hand, Ctnnb1 signaling is necessary for osteoblast and osteocyte expression of OPG and induce osteogenesis [25]. Ctnnb1 inhibits adipogenesis in mesenchymal stem cell lineage, apoptosis in osteoblasts and differentiation of monocytes to osteoclasts [26]. Similar to OPG/RANK-L ratio, we observed that maternal LF-HCD during gestation and lactation lead to Runx2 and Ctnnb1 up-regulation compared with the HF-LCD. At protein level, serum OPG/RANK-L ratio was significantly higher in the offspring born from LF-HCD-fed mothers than HF-LCD group. Adjusted for the diets, OPG/RANK-L expression ratio was higher in female than male offspring, but sex had no significant effect on the expression of other target genes. Previous studies mentioned that 17β-estradiol enhances OPG gene expression and protein secretion through a transcriptional mechanism in MSCs [27, 28]. PPARγ2 is the dominant regulator of adipogenesis which its activation favors the differentiation of MSCs into adipocytes rather than osteoblasts [29]. Our results showed that maternal HF-LCD during gestation and lactation lead to PPARγ2 up-regulation in the bone of offspring, compared with LF-HCD. Accumulating data suggest that excessive fat mass is detrimental to bone [30, 31]. Although, there is a substantial body of evidence that maternal high calorie/high fat diet affect bone metabolism, growth, remodeling, and turnover [32], currently there is no evidence available on the effects of maternal isocaloric diet containing different amounts of fat and carbohydrate on bone.

LPL and FASN are adipogenic and lipogenic markers which are quantitatively important for synthesis and deposition of fatty acids in adipose tissues. Studies have shown that LPL and FASN over-expression result in higher fat mass and more insulin resistance [33, 34]. Leptin and adiponectin are the fat-derived hormones which effect centrally or peripherally on bone, that antagonize each other. Studies have shown that hypothalamic pathways of these adipokins are stronger than peripheral effects [35]. Leptin suppresses osteoblast activity and increases osteoclast numbers via RANK-L and b2-adrenergic receptor (Adrb2) [36, 37]. Adiponectin acts directly in osteoblasts to prevent their proliferation and increase their apoptosis in a PI3-kinase-FoxO1-dependent manner in young ages. RANK-L expression was increased more than 10-folds after stimulation by even low amounts of adiponectin or its globular domain. These results identifying the osteoblast and RANK-L as adiponectin target cell and gene implied that this hormone should inhibit bone mass accrual by favoring bone resorption. Over the time, by aging, this local effect obscured through sympathetic nervous system inhibition [38]. Results of our study are in agreement with these concepts. Adiponectin, Lep, FASN and LPL genes expression were increased in offspring born from the HF-LCD compared with the LF-HCD-fed mothers. In opposite OPG and Ctnnb1 as the markers of osteogenesis were decreased. Then, adiponectin signals back into osteoblasts and hamper their proliferation and favor their apoptosis in young age (6 weeks old). At protein level, serum leptin and adiponectin were significantly higher in the female offspring of HF-LCD-fed mothers than LF-HCD. In male offspring, serum leptin level was significantly higher in the HF-LCD-fed group. These results suggest that alterations in gene expression occur at protein level which is functional.

In summary, in the same calorie contents, LF-HCD is preferred to HF-LCD for maternal consumption during gestation and lactation due to increases in osteogenesis and decreases in adipogenesis in the next generation. Then, not only calorie content but also, macronutrient distribution in maternal diet during gestation and lactation is important. Therefore, our hypothesis mentioned at the beginning of the essay will be accepted.

There are some limitations to our data, most importantly, the fact that the background features of maternal mice fed with LF-HCD vs. HF-LCD should be provided. Skeletal and adipose tissue gene expression were assessed only at adolescence, even though periodic measures from birth until aging, is recommended. This study does not directly measure whether maternal LF-HCD and HF-LCD during gestation and lactation induce epigenetic changes on bone and fat mass of offspring or not. In future studies, direct assessment of epigenetic changes such as histone modification or DNA methylation of the mentioned target genes will allow us to test the hypothesis that perinatal programming of the skeleton occurs through epigenetic mechanisms. Additionally, genes expression was normalized against GAPDH as the housekeeping gene. Other housekeeping genes other than GAPDH are suggested for future studies. A free-fed group along with the pair-fed groups is also suggested for the future. This is an animal model; therefore, the effects of the diets cannot be directly transposed to human. These studies provide indications of the possible effects of such dietary manipulations on fat and skeletal physiology that may suggest future insights for research of underlying mechanisms.

Larger time-point studies that will include evaluation of the developing bone by using in vivo micro-CT as well as bone strength testing, bone mineral density, histology and histomorphometry and measurement of bone and adipose tissue will lead to further insights about post-translational modifications.

## Conclusions

Our results suggest that in the same calorie contents, high fat-low carbohydrate composition in maternal diet during gestation and lactation result a significant changes in bone formation and retroperitoneal fat gene expressions that modulate early-life events to potentiate protection from the risk of bone disorders in female and male offspring adult life. Findings from this study suggest that both the calorie content and macronutrient distribution effect on the next generation. Further research is warranted to explore the exact mechanisms in this process.

## Abbreviations

Ctnnb1: Beta-catenin
FASN: Fatty acid synthase
HF-LCD: High fat-low carbohydrate diet
LF-HCD: Low fat-high carbohydrate diet
LPL: Lipoprotein lipase
MSC: Mesenchymal stem cell
OPG: Osteoprotegrin
PPARɤ: Peroxisome proliferator-activated receptor gamma
RANK-L: Receptor activator of nuclear factor NF-κB ligand
